# Global incidence trends of early-onset colorectal cancer and related exposures in early-life: an ecological analysis based on the *GBD 2019*

**DOI:** 10.3389/fpubh.2024.1367818

**Published:** 2024-06-20

**Authors:** Ziyang Wang, Weiyuan Yao, Weimiao Wu, Junjie Huang, Yanlei Ma, Chen Yang, Jufang Shi, Jiongxing Fu, Yingying Wang, Martin C. S. Wong, Wanghong Xu

**Affiliations:** ^1^Global Health Institute, Fudan University School of Public Health, Shanghai, China; ^2^Jockey Club School of Public Health and Primary Care, Faculty of Medicine, The Chinese University of Hong Kong, Sha Tin, Hong Kong SAR, China; ^3^Department of Colorectal Surgery, Fudan University Shanghai Cancer Center, Shanghai, China; ^4^Centre for Disease Control & Prevention in Pudong New Area of Shanghai, Shanghai, China; ^5^Office of Cancer Screening, National Cancer Centre/National Clinical Research Centre for Cancer/Cancer Hospital, Chinese Academy of Medical Sciences and Peking Union Medical College, Beijing, China

**Keywords:** incidence, early-onset colorectal cancer, early life exposure, ecological study, *GBD 2019*

## Abstract

**Background:**

The incidence of early-onset colorectal cancer (EOCRC) is increasing globally. This study aims to describe the temporal trends of incidence and explore related risk exposures in early-life at the country level based on the *GBD 2019*.

**Methods:**

Data on the incidence and attributable risk factors of EOCRC were obtained from the *GBD 2019*. Temporal trends of age-standardized incidence were evaluated by average annual percentage change (AAPC). Early-life exposures were indicated as summary exposure values (SEV) of selected factors, SDI and GDP *per capita* in previous decades and at ages 0–4, 5–9, 10–14 and 15–19 years. Weighted linear or non-linear regressions were applied to evaluate the ecological aggregate associations of the exposures with incidences of EOCRC.

**Results:**

The global age-standardized incidence of EOCRC increased from 3.05 (3.03, 3.07) to 3.85 (3.83, 3.86) per 100,000 during 1990 and 2019. The incidence was higher in countries with high socioeconomic levels, and increased drastically in countries in East Asia and Caribbean, particularly Jamaica, Saudi Arabia and Vietnam. The GDP *per capita*, SDI, and SEVs of iron deficiency, alcohol use, high body-mass index, and child growth failure in earlier years were more closely related with the incidences of EOCRC in 2019. Exposures at ages 0–4, 5–9, 10–14 and 15–19 years were also associated with the incidences, particularly for the exposures at ages 15–19 years.

**Conclusion:**

The global incidence of EOCRC increased during past three decades. The large variations at regional and national level may be related with the distribution of risk exposures in early life.

## Introduction

The incidence of early-onset colorectal cancer (EOCRC) (diagnosed before age 50 years) has been increasing since the mid-1990s in the United States (1), and became the leading cause of cancer incidence and mortality among young adults in 2017 (2). The rising incidence of EOCRC was also well documented in other countries in recent years (3, 4), especially in countries with high Human Development Index (HDI) (5). Several studies described the global upward trends of the incidence and mortality of EOCRC based on the Global Burden of Disease, Injuries and Risk factors 2019 (*GBD 2019*) (4, 6, 7), and the Global Cancer Observatory (*GLOBOCAN*) (8), arousing extensive research interests on the new challenge for population health. As EOCRC is more likely to present adverse histologic features and develop metastatic diseases (9), the increased EOCRC has caused severe financial and life years loss in young patients (10), and is becoming a substantial public health problem globally.

The upward trend of the global incidence of EOCRC indicate substantial changes in risk exposures. It is essential to identify risk factors of EOCRC and therefore stem the tide either by decreasing risk exposures (11) or through risk-stratification screening in young populations (12). However, the risks and preventive factors for EOCRC have not been comprehensively reported. Most previous studies mainly focused on demographic and lifestyle factors, family history of CRC and specific comorbidities during adulthood (13–15). As colorectal carcinogenesis is a typically long-term process lasting for decades, exposure to risk factors in early-life, i.e., from peri-conception to 20 years old, may contribute to the development of EOCRC (16). The hypothesis is supported by a report of Murphy and colleagues (17), in which the age-specific incidence of EOCRC was observed to increase across successive birth cohorts after 1960 in the United States, indicating a significant birth cohort effect and suggesting that early life is an important window of susceptibility for EOCRC.

Nevertheless, there was a paucity of epidemiological evidence on early-life risk factors with EOCRC, probably due to the difficulty in exposure data collection. Based on a large-scale population-based case–control study, Li et al. (18) observed significant associations of body mass index (BMI) and obesity at 20 years with the risk of CRC among subjects aged younger than 55 years. However, a nested case–control study based on the UK Biobank cohort did not find significant associations between multiple factors in early-life and the risk of EOCRC (19). A study based on the Nurses’ Health Study reported a compelling association between obesity at age 18 years and EOCRC in women (20).

In this study, we made use of the *GBD 2019* data to describe the incidence of EOCRC and its temporal trends at global, regional and national levels. In addition, we speculate that the large variations of incidence at regional and national level may be related with the distribution of risk exposures in early life, and test the hypothesis by evaluating the aggregate associations of exposures in previous decades or during childhood and adolescence with the incidence of EOCRC at the country level.

## Materials and methods

### Data sources

The *GBD 2019* is a collaborative multinational study providing full time-series estimates of incidence, prevalence, mortality, years lived with disability (YLDs), years of life lost (YLLs), and disability adjusted Life Years (DALYs) for 369 diseases and injuries by sex and age groups from 1990 to 2019 for 204 countries and territories under 7 super-regions, 21 regions, and 5 social demographic index (SDI) regions (low SDI, low-middle SDI, middle SDI, high-middle SDI, and high SDI) (21, 22). It also included 87 risk factors that are broadly categorised into three groups: (1) environmental and occupational, (2) behavioral, and (3) metabolic.

We synthesised the *GBD 2019* data to assess the incidence and secular trends of CRC in young populations (age < 50 years) and explored the related factors in early life. Specifically, we extracted SDI, age-specific incidence of EOCRC, and summary exposure values (SEV) of risk factors in the populations at country/territory level from the website of https://vizhub.healthdata.org/gbd-results/ using the Global Heath Data Exchange (GHDx) Tool (23). We also downloaded the gross domestic product (GDP) data from the World Bank Databank.^1^ We further obtained the sex-specific data from *GBD 2019*, if sex stratified information is available.

### Definition of EOCRC

CRC was coded as C18-21, D01.0-D01.2, or D12-D12.9 in the 10th revision of the International Classification of Diseases (ICD-10) (24). In this study, we defined EOCRC as CRC diagnosed before 50 years. Using the GHDx tool and by selecting the term of “colon and rectum cancer” as the “cause,” and “Incidence” as the “measure,” we obtained age-specific incidence of EOCRC for ten 5-year age groups (0–4, 5–9, 10–14, 15–19, 20–24, 25–29, 30–34, 35–39, 40–44 and 45–49 years). The methodology adopted by the *GBD 2019* to estimate incidence of CRC has been described previously (25). Briefly, the data derived from vital registration systems, vital registration samples, verbal autopsy, and cancer registry were used to calculate mortality-to-incidence ratios. Then a spatiotemporal Gaussian process regression was applied to model mortality-to-incidence ratios for all combinations of age, sex, year, and location with incidence data from cancer registries and mortality data from cancer registries or high-quality vital statistics registries. Estimates of mortality obtained with mortality-to-incidence ratios were combined with vital registration and verbal autopsy mortality data and used as inputs in cancer type and sex-specific Cause of Death Ensemble models (CODEm) (26). And then incidence of specific cancer (e.g., CRC) were estimated by dividing the mortality estimates by the corresponding mortality-to-incidence ratios for each cancer type by gender, age group, location and calendar year (21). Strength of the model ensured the comparability of the data provided across periods and regions. Evidently, the quality of the cancer registry and the vital statistic data may have been improved over time, and was found higher in high-SDI countries than those with low SDI level (24).

### Early-life risk exposures

SEV was used in *GBD 2019* to summarise the exposure distribution of various risk factors in a population, which were provided in 5-year age groups (0–4, 5–9, 10–14, 15–19, etc.). The SEV for a risk factor was defined as a measure of a population’s exposure to the factor that takes into account the extent of exposure by risk level and the severity of that risk’s contribution to disease burden. The SEV is effectively excess risk-weighted prevalence, which allows for comparisons across different types of exposures (22). The equation and detailed information for SEV calculation were provided as Supplementary content 1.

The extracted country-level GDP *per capita*, SDI, and age-specific SEVs for all ten behavioral and metabolic factors in the populations less than 20 years were regarded as candidate factors, which included short gestation, low birth weight, exposure to suboptimal breastfeeding, child growth failure, childhood sexual abuse and bullying, alcohol abuse, drug use, intimate partner violence, iron deficiency, and high body mass index (BMI). We further identified the potential risk factors according to the correlations between the SEVs of the candidate factors and ASIR of EOCRC at global level over 1990 and 2019 (Supplementary Figure S1) and at the nation level in 1990, 2000, 2010 and 2019 (Supplementary Figure S2). Finally, we included the factors consistently correlated with ASIR of EOCRC and those previously reported as risk factors for EOCRC (18–20, 27), i.e., child growth failure, suboptimal breastfeeding, alcohol use, high BMI and iron deficiency, in this analysis.

We further evaluated the risk exposures in early-life through two approaches. First, by assuming that people aged 0–19 years in 1990, 2000 and 2010 in each country or territory were the same people aged 30–49, 20–39 and 10–29 years in 2019, we treated the SEVs of selected factors in a specific age group in 1990, 2000 and 2010 as risk exposures in early life for corresponding people in 2019. For example, for suboptimal breastfeeding available at 0–4 years in *GBD 2019*, we evaluated the association between its SEV in 1990 and the incidence of EOCRC among people aged 30–34 years in 2019, and so on; for high BMI available for 0–19 years, we estimated the association of age-standardized SEV (aged 0–19 years) in 1990 with age-standardized incidence rate (ASIR) of EOCRC in people aged 30–49 years in 2019, and so forth.

Second, we used the age-specific SEVs of selected factors at four period (i.e., 0–4, 5–9, 10–14 and 15–19 years) as risk exposure windows in early life. As shown in Supplementary Figure S3, for people aged 20–24 years in 2019, their exposure to risk factors at 10–14 years were extracted from year 2009; for people aged 25–29 years in 2019, their exposures at age 10–14 years were extracted from the data in 2004; in other words, the exposures at 10–14 years were extracted from the year of 1999, 2004, 2009, 2014 and 2019 for people at ages of 35–39, 30–34, 25–29, 20–24, 15–19, and 10–14 years in 2019, respectively. The extracted SEVs for each exposure age window were further weighted for the association evaluation with ASIR of EOCRC in 2019.

Similarly, the SDI and the GDP *per capita* for each country or territory from 1990 to 2019 were used to gain deep understanding of the associations of socioeconomic factors during childhood and adolescence with the incidence of EOCRC in 2019.

### Statistical analysis

Age-standardised incidence of EOCRC and SEVs of risk factors were estimated based on the GBD world standard population using a direct method (28). Average annual percentage changes (AAPCs) and 95% confidence intervals (CIs) of the age-standardized incidence, GDP *per capita,* SDI and SEVs were calculated using Joinpoint regression analysis to estimate the temporal patterns (29).

Associations of incidence with risk exposures were examined at national level by weighted linear or non-linear regression, mainly through the local weighted scatter plot regression (LOWESS). For sex-specific data, we performed stratified analyses to evaluate the associations in the male and female populations. We also conducted stratified analyses by SDI levels of the countries to demonstrate the potential impact of quality of registration on the association evaluations. Multi-variable analyses were further performed to evaluate the adjusted associations. Variance Inflation Factor (VIF) was used to investigate for multi-collinearity of the risk factors during a same period or at a same age window. We did not observe a significant collinearity and any substantially changed associations after mutual adjustment (Supplementary Table S1). We therefore presented the unadjusted associations as the main results.

Considering that the American Cancer Society recommended to lower the age of initial screening from 50 to 45 years in 2018 (30), we ran a sensitivity analysis on temporal patterns in incidence using the data of the United States by including or excluding the age-group of 45–49 years in the country, respectively.

All data analyses were performed using R 4.2.0., mainly R package of “epitools.” Joinpoint Regression Program 4.9.1.0 was used to evaluate the secular trends of EOCRC and risk exposures. *p* value less than 0.05 was considered statistically significant.

### Patient and public involvement statement

The GBD study is a global collaborative scientific effort involving more than 7,500 people from about 150 countries. No patients were involved in setting the specific research question, collecting and analysing the data, interpreting the results, or writing up the manuscript. The research findings will be disseminated to the wider community by press releases, social media platforms, presentations at international fora, reports to relevant government agencies and academic societies.

## Results

### Incidence and temporal pattern of EOCRC

The global age-standardised incidence of EOCRC reached 3.85 (95%CI, 3.83–3.86) per 100,000 in 2019, and was higher in the male [4.64 (95%CI, 4.61–4.66) per 100,000] than the females [3.05 (95%CI, 3.03–3.07) per 100,000]. We also observed marked increases in incidence from 1990 to 2019, with an AAPC of 1.0% (95%CI, 0.8–1.2) over the decades (Table 1).

**Table 1:** Age-standardized incidence of early-onset colorectal cancer in 2019 and AAPC during 1990–2019 at global and regional level.

	Age-standardized incidence (95% confidence interval), 1/100,000			Average annual percentage change (95% confidence interval), %		
	Overall	Male	Female	Overall	Male	Female
Global	3.85 (3.83, 3.86)	4.64 (4.61, 4.66)	3.05 (3.03, 3.07)	1.0 (0.8, 1.2)	1.7 (1.5, 1.9)	0.3 (0.1, 0.5)
By region						
Eastern Sub-Saharan Africa	1.36 (1.31, 1.41)	1.43 (1.36, 1.51)	1.28 (1.22, 1.35)	0.5 (0.3, 0.8)	0.8 (0.5, 1.0)	0.2 (−0.2, 0.7)
High-income Asia Pacific	5.80 (5.68, 5.93)	6.57 (6.39, 6.75)	5.00 (4.84, 5.17)	−0.1 (−0.4, 0.2)	−0.1 (−0.5, 0.2)	0.0 (−0.7, 0.6)
East Asia	6.95 (6.90, 6.99)	9.57 (9.49, 9.64)	4.22 (4.17, 4.27)	2.8 (2.3, 3.4)	3.7 (3.0, 4.3)	1.3 (1.1, 1.6)
Australasia	6.78 (6.44, 7.13)	7.06 (6.57, 7.58)	6.51 (6.05, 6.99)	0.5 (0.3, 0.8)	0.6 (0.3, 0.9)	0.5 (0.3, 0.6)
Eastern Europe	5.17 (5.06, 5.28)	5.44 (5.28, 5.60)	4.92 (4.77, 5.07)	0.9 (0.0, 1.9)	1.1 (0.1, 2.1)	0.8 (−0.1, 1.7)
Southern Sub-Saharan Africa	3.07 (2.93, 3.21)	3.46 (3.26, 3.68)	2.68 (2.50, 2.87)	1.2 (−0.7, 3.1)	0.9 (−1.1, 2.9)	1.4 (−0.5, 3.5)
Central Sub-Saharan Africa	1.10 (1.02, 1.18)	1.22 (1.10, 1.34)	0.98 (0.88, 1.10)	0.0 (−0.4, 0.5)	−0.1 (−0.5, 0.4)	0.1 (−0.4, 0.5)
South Asia	1.36 (1.34, 1.38)	1.24 (1.22, 1.27)	1.47 (1.44, 1.50)	1.2 (1.0, 1.4)	1.4 (1.2, 1.6)	1.1 (0.7, 1.5)
Central Europe	5.01 (4.87, 5.16)	5.75 (5.53, 5.97)	4.26 (4.08, 4.46)	0.4 (0.2, 0.6)	0.5 (0.3, 0.8)	0.3 (0.1, 0.5)
Central Asia	2.43 (2.32, 2.55)	2.68 (2.51, 2.86)	2.19 (2.03, 2.35)	−0.9 (−1.4, −0.3)	−1.0 (−1.6, −0.3)	−0.8 (−1.2, −0.3)
Western Europe	4.96 (4.89, 5.04)	5.23 (5.13, 5.35)	4.69 (4.58, 4.79)	0.1 (−0.2, 0.3)	0.2 (−0.2, 0.5)	0.1 (−0.1, 0.3)
Oceania	1.87 (1.59, 2.18)	2.12 (1.72, 2.60)	1.60 (1.25, 2.03)	0.3 (0.2, 0.5)	0.4 (0.2, 0.5)	0.3 (0.2, 0.4)
Southeast Asia	3.50 (3.45, 3.55)	4.11 (4.03, 4.19)	2.90 (2.83, 2.96)	1.3 (1.1, 1.6)	1.6 (1.4, 1.8)	1.0 (0.8, 1.2)
Andean Latin America	3.13 (2.97, 3.29)	3.53 (3.29, 3.78)	2.74 (2.53, 2.96)	2.7 (1.5, 4.0)	3.1 (1.9, 4.3)	2.3 (1.0, 3.7)
Western Sub-Saharan Africa	1.04 (1.00, 1.08)	1.15 (1.09, 1.21)	0.94 (0.89, 1.00)	0.9 (0.7, 1.1)	1.0 (0.8, 1.1)	0.8 (0.6, 1.0)
Tropical Latin America	3.10 (3.02, 3.18)	3.19 (3.07, 3.31)	3.01 (2.90, 3.12)	1.6 (1.2, 2.1)	1.7 (1.3, 2.1)	1.4 (1.0, 1.8)
Southern Latin America	4.26 (4.08, 4.44)	4.71 (4.45, 4.99)	3.83 (3.59, 4.07)	1.4 (1.2, 1.5)	1.4 (1.3, 1.6)	1.3 (1.2, 1.5)
North Africa and Middle East	2.26 (2.21, 2.30)	2.60 (2.54, 2.67)	1.87 (1.81, 1.92)	1.0 (0.4, 1.5)	1.3 (0.6, 2.0)	0.5 (0.1, 1.0)
High-income North America	6.86 (6.76, 6.96)	7.45 (7.30, 7.60)	6.28 (6.14, 6.41)	0.8 (0.4, 1.2)	0.8 (0.4, 1.2)	1.0 (0.8, 1.2)
Caribbean	3.46 (3.27, 3.66)	3.64 (3.36, 3.93)	3.29 (3.03, 3.57)	1.0 (0.8, 1.2)	1.1 (0.9, 1.3)	0.8 (0.6, 1.0)
Central Latin America	3.05 (2.97, 3.13)	3.38 (3.26, 3.50)	(2.64, 2.85)	2.3 (2.1, 2.5)	2.5 (2.2, 2.7)	2.1 (1.9, 2.3)
By SDI						
High SDI	5.85 (5.79, 5.90)	6.40 (6.32, 6.48)	5.26 (5.19, 5.33)	0.5 (0.2, 0.8)	0.6 (0.4, 0.7)	0.4 (0.2, 0.7)
High-middle SDI	5.62 (5.57, 5.66)	7.07 (7.01, 7.14)	4.11 (4.06, 4.16)	1.7 (1.4, 2.0)	2.3 (1.9, 2.7)	0.8 (0.4, 1.2)
Middle SDI	3.89 (3.86, 3.91)	4.98 (4.94, 5.03)	2.78 (2.75, 2.81)	2.1 (1.8, 2.3)	2.8 (2.5, 3.0)	1.2 (0.8, 1.5)
Low-middle SDI	2.01 (1.99, 2.04)	2.11 (2.07, 2.15)	1.91 (1.88, 1.95)	1.4 (1.2, 1.6)	1.8 (1.6, 1.9)	1.0 (0.8, 1.2)
Low SDI	1.26 (1.23, 1.29)	1.25 (1.21, 1.29)	1.27 (1.23, 1.31)	0.6 (0.5, 0.7)	0.7 (0.6, 0.8)	0.5 (0.4, 0.6)

At the regional level, the incidence was the highest in East Asia [6.95 (95%CI, 6.90–6.99) per 100,000], followed by high-income countries in North America [6.86 (6.76, 6.96) per 100,000], Australasia [6.78 (6.44, 7.13) per 100,000] and high-income Asia Pacific countries/regions [5.80 (5.68, 5.93) per 100,000]. The incidence increased from 1990 to 2019 in most regions, with the exception of central Asia [AAPC: −0.9% (−1.4,-0.3)], high-income Asia Pacific [AAPC: −0.1% (−0.4, 0.2)], central sub-Saharan African [AAPC: 0.0% (−0.4, 0.5)], Western Europe [AAPC: 0.1% (−0.2, 0.3)] and Southern Sub-Saharan Africa [AAPC: 1.2% (−0.7, 3.1)]. The upward pattern was more pronounced in East Asia [AAPC: 2.8% (95%CI, 2.3, 3.4)], Andean Latin America [AAPC: 2.7% (1.5, 4.0)], and Central Latin America [AAPC: 2.3% (2.1, 2.5)]. Further analysis by SDI demonstrated increasing trends of the incidences in all regions, and the highest incidence in high-SDI regions and the highest AAPC in middle SDI regions.

**Figure 1:**
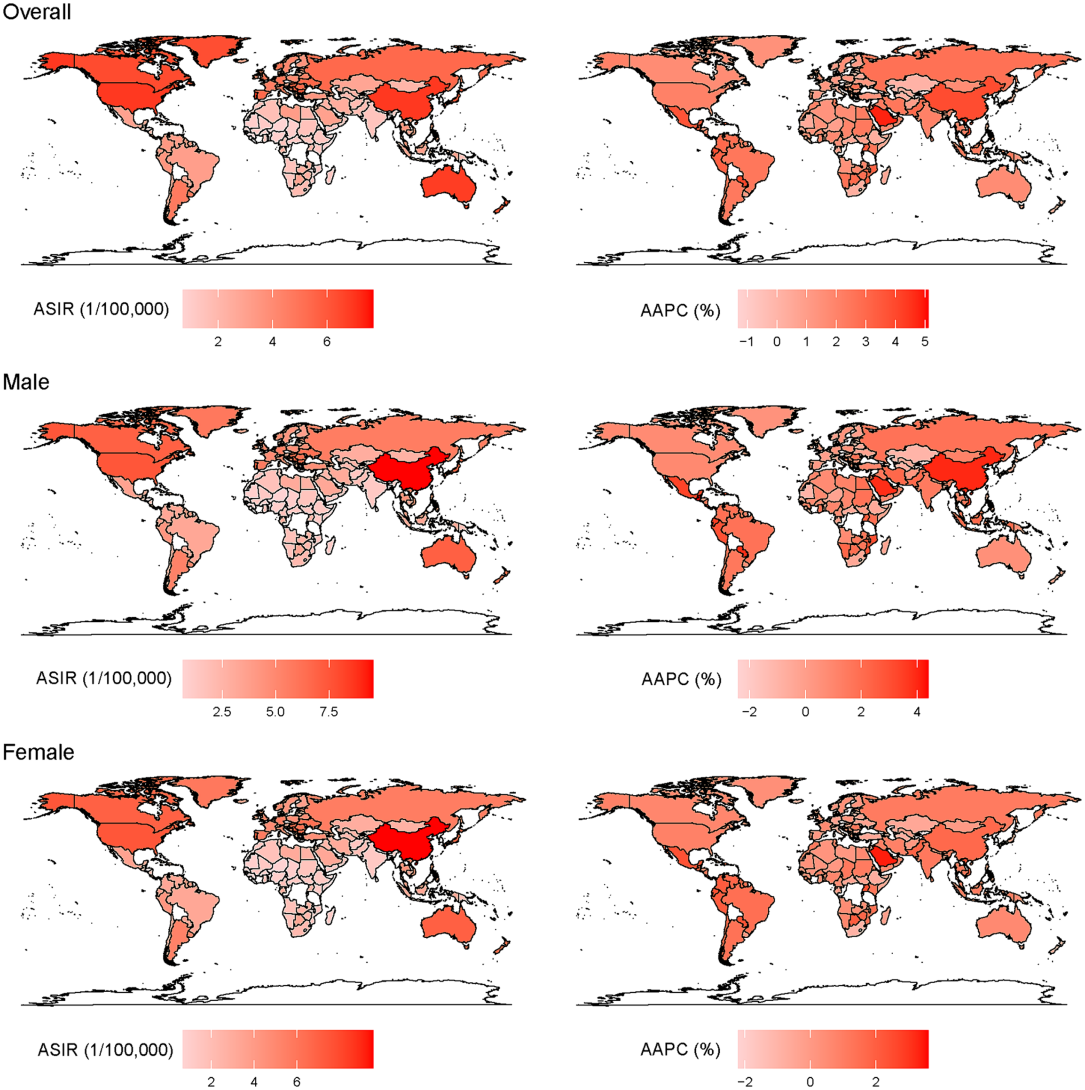
Age-standardized incidence of EOCRC (left) in 2019 and AAPC (right) from 1990 to 2019 at the country level.

The incidence of EOCRC in 2019 at the country/territory level are presented in Figure 1. The highest incidence was observed in Taiwan, a province of China [32.73 (31.46, 34.04) per 100, 000], followed by Monaco [19.77 (2.24, 86.45) per 100, 000], Portugal [18.59 (17.14, 20.15) per 100, 000], the United States [18.09 (17.81, 18.38) per 100, 000], and Australia [17.48 (15.61, 18.49) per 100, 000]. The incidence increased most rapidly in Jamaica [AAPC: 5.1% (3.2, 7.0)], Saudi Arabia [AAPC: 4.8% (4.6, 5.1)] and Vietnam [AAPC: 4.4% (4.3, 4.5)] overall; in Guatemala for men [AAPC: 4.4% (3.8, 4.9)] and Jamaica for women [AAPC: 3.6% (2.6, 4.6)].

Sensitivity analysis on the temporal pattern of incidence based on the data of the United States was conducted by including or excluding subgroup of 45–49 years who might have received mass screening for CRC. As shown in Supplementary Figure S4, two small peaks in incidence were observed in populations aged 45–49 years around 2002 and 2011, indicating the influence of CRC screening. However, no substantial change was observed in overall trend before and after excluding the subgroup.

### Exposures to selected factors and temporal trends

As shown in Supplementary Figure S5, the national level of GDP *per capita* and SDI remained at the highest level in Monaco, Liechtenstein, and Switzerland in 1990, 2000, 2010 and 2019, and increased more or less in all countries over the four calendar years. The GDP *per capita* increased most in Equatorial Guinea during 1990 and 2019 [AAPC of 17.5% (14.0, 21.1)], followed by Vietnam [AAPC of 13.1% (11.8, 14.5)] and China [AAPC of 12.7% (11.8, 13.6)], while the SDI increased most in Equatorial Guinea [AAPC of 4.2% (4.1, 4.4)], Mozambique [AAPC of 3.3% (3.2, 3.4)], and Uganda [AAPC of 3.1% (3.0, 3.2)].

Table 2 presents the SEVs of suboptimal breastfeeding, child growth failure, iron deficiency, alcohol use and high BMI in 1990, 2000, 2010 and 2019. Alcohol consumption and high BMI were observed to increase over the four time points, while a decreasing trend was observed for suboptimal breastfeeding, iron deficiency and child growth failure. Generally, the male populations were more likely to expose to alcohol consumption and high BMI than the females, while the female populations were more likely to be suboptimal breastfed and suffer from child growth failure. However, the temporal trends of the exposures were similar in both sexes. Further analysis by SDI demonstrated higher child growth failure and iron deficiency in low-SDI regions, and higher suboptimal breastfeeding, alcohol use and high BMI in high-SDI regions. The highest AAPC of high BMI was observed in regions with middle SDI.

**Table 2:** Global summary exposure values of selected risk factors and average annual percent changes during the period of 1990 and 2019.

	Summary exposure value (95% confidence interval), %				Average annual percentage change (95% confidence interval), %
	Year 1990	Year 2000	Year 2010	Year 2019	
**Suboptimal breastfeeding**	21.85 (20.44, 23.19)	20.73 (19.06, 22.03)	20.10 (18.29, 21.39)	19.13 (17.25, 20.44)	−0.4 (−0.6, −0.3)
By sex					
Male	15.19 (13.67, 17.19)	14.06 (12.63, 15.92)	13.43 (12.06, 15.92)	12.61 (11.40, 14.19)	−0.6 (−0.8, −0.5)
Female	28.77 (26.67, 29.50)	27.73 (25.08, 28.53)	27.10 (24.05, 28.03)	25.98 (22.70, 27.04)	−0.3 (−0.4, −0.2)
By SDI					
High SDI	29.67 (19.97, 42.44)	29.03 (19.45, 41.68)	28.90 (19.34, 41.53)	28.50 (19.01, 41.06)	−0.1 (−0.2, 0.0)
High-middle SDI	25.08 (16.24, 37.00)	24.58 (15.84, 36.40)	24.66 (15.90, 36.50)	22.91 (14.51, 34.41)	−0.3 (−0.8, 0.2)
Middle SDI	22.49 (14.18, 33.90)	21.99 (13.78, 33.30)	21.44 (13.35, 32.63)	19.75 (12.02, 30.59)	−0.4 (−0.9, 0.1)
Low-middle SDI	18.78 (11.27, 29.40)	17.45 (10.24, 27.77)	16.89 (9.82, 27.09)	16.72 (9.69, 26.88)	−0.4 (−0.8, 0.0)
Low SDI	17.68 (10.42, 28.06)	17.26 (10.10, 27.54)	16.72 (9.69, 26.88)	16.17 (9.27, 26.19)	−0.3 (−0.4, −0.2)
**Child growth failure**	4.93 (4.41, 5.57)	4.86 (4.33, 5.48)	4.21 (3.70, 4.78)	3.53 (3.00, 4.10)	−1.1 (−2.4, 0.2)
By sex					
Male	4.81 (4.28, 5.45)	4.76 (4.23, 5.37)	4.11 (3.59, 4.67)	3.42 (2.91, 3.98)	−1.1 (−2.5, 0.3)
Female	5.06 (4.54, 5.71)	4.97 (4.43, 5.59)	4.32 (3.80, 4.90)	3.64 (3.11, 4.23)	−1.1 (−2.3, 0.2)
By SDI					
High SDI	0.67 (0.00, 4.98)	0.61 (0.00, 4.89)	0.59 (0.00, 4.84)	0.57 (0.00, 4.81)	−0.5 (−1.0, −0.1)
High-middle SDI	1.99 (0.24, 7.20)	2.03 (0.25, 7.27)	1.87 (0.20, 7.02)	1.61 (0.13, 6.60)	−0.7 (−1.9, 0.6)
Middle SDI	3.47 (0.83, 9.47)	3.29 (0.75, 9.19)	2.99 (0.62, 8.76)	2.52 (0.42, 8.04)	−1.0 (−1.9, −0.1)
Low-middle SDI	8.34 (3.68, 16.21)	7.40 (3.07, 14.97)	5.93 (2.16, 12.96)	4.81 (1.52, 11.39)	−1.9 (−2.7, −1.0)
Low SDI	7.87 (3.37, 15.59)	7.42 (3.08, 14.99)	6.17 (2.30, 13.29)	5.16 (1.72, 11.90)	−1.4 (−2.5, −0.4)
**Iron deficiency**	19.21 (16.92, 21.43)	18.58 (16.45, 20.61)	17.92 (15.88, 19.87)	17.42 (15.26, 19.44)	−0.3 (−0.4, −0.3)
By SDI					
High SDI	10.42 (6.44, 15.95)	9.61 (5.81, 14.98)	9.14 (5.44, 14.40)	8.89 (5.25, 14.10)	−0.5 (−0.9, −0.2)
High-middle SDI	14.82 (9.97, 21.21)	13.87 (9.20, 20.08)	12.53 (8.11, 18.49)	11.86 (7.58, 17.70)	−0.8 (−1.0, −0.6)
Middle SDI	17.60 (12.27, 24.45)	16.39 (11.27, 23.05)	15.33 (10.39, 21.80)	14.54 (9.75, 20.89)	−0.7 (−0.7, −0.6)
Low-middle SDI	25.10 (18.64, 33.07)	23.67 (17.42, 31.45)	21.93 (15.93, 29.45)	20.12 (14.39, 27.38)	−0.8 (−1.0, −0.5)
Low SDI	30.00 (22.90, 38.62)	28.89 (21.93, 37.37)	26.56 (19.90, 34.73)	24.69 (18.29, 32.61)	−0.7 (−1.0, −0.3)
**Alcohol use**	2.95 (1.68, 4.77)	3.37 (1.87, 5.57)	3.72 (2.06, 6.14)	4.28 (2.30, 7.15)	1.3 (0.9, 1.6)
By sex					
Male	4.21 (2.41, 6.75)	4.92 (2.73, 8.06)	5.50 (3.02, 9.08)	6.49 (3.49, 10.82)	1.5 (1.1, 1.8)
Female	1.64 (0.91, 2.75)	1.73 (0.94, 2.94)	1.82 (0.98, 3.10)	1.93 (1.01, 3.31)	0.6 (0.5, 0.6)
By SDI					
High SDI	10.14 (7.26, 13.78)	11.62 (8.52, 15.47)	9.76 (5.93, 15.16)	10.55 (6.53, 16.13)	0.0 (−2.0, 2.1)
High-middle SDI	6.97 (4.62, 10.08)	7.30 (4.90, 10.47)	6.55 (3.50, 11.19)	7.34 (4.07, 12.20)	0.0 (−1.1, 1.2)
Middle SDI	2.41 (1.14, 4.49)	3.10 (1.62, 5.37)	2.61 (0.87, 5.99)	3.03 (1.12, 6.60)	0.6 (−2.2, 3.5)
Low-middle SDI	1.35 (0.46, 3.07)	1.91 (0.80, 3.82)	1.43 (0.28, 4.29)	1.62 (0.36, 4.58)	0.4 (−4.1, 5.1)
Low SDI	0.69 (0.13, 2.11)	1.21 (0.38, 2.86)	1.13 (0.17, 3.83)	1.19 (0.19, 3.93)	1.5 (−3.7, 7.0)
**High body mass index**	8.77 (7.02, 12.01)	11.06 (9.03, 14.90)	13.77 (11.47, 18.01)	16.80 (14.08, 21.52)	2.3 (2.2, 2.3)
By sex					
Male	8.30 (6.02, 13.36)	10.58 (7.87, 16.11)	13.32 (10.18, 19.48)	16.64 (12.92, 23.53)	2.1 (1.9, 2.3)
Female	9.26 (6.58, 14.29)	11.57(8.43, 17.03)	14.26 (10.72, 20.53)	16.98 (12.90, 23.70)	2.4 (2.3, 2.5)
By SDI					
High SDI	17.38 (13.53, 21.98)	23.24 (18.76, 28.49)	27.06 (22.20, 32.67)	28.84 (23.81, 34.66)	1.7 (0.1, 3.2)
High-middle SDI	10.96 (7.95, 14.74)	13.67 (10.28, 17.82)	18.17 (14.23, 22.87)	21.97 (17.59, 27.15)	2.5 (2.0, 2.9)
Middle SDI	8.25 (5.67, 11.60)	11.29 (8.23, 15.11)	15.05 (11.49, 19.38)	19.16 (15.08, 24.03)	2.9 (2.6, 3.2)
Low-middle SDI	5.65 (3.56, 8.53)	7.27 (4.87, 10.45)	9.54 (6.76, 13.10)	13.04 (9.71, 17.17)	3.0 (2.3, 3.7)
Low SDI	6.33 (4.10, 9.34)	7.20 (4.81, 10.37)	8.78 (6.12, 12.22)	11.37 (8.28, 15.27)	2.1 (1.0, 3.2)

Summary exposure value of child growth failure defined based on the WHO 2006 growth standards for children 0–59 months using *Z*-scores derived from an international reference population.

As profiled in Supplementary Figure S6, alcohol use, high BMI and suboptimal breastfeeding were higher in the United Kingdom, the United States and Monaco, which are countries with high GDP *per capita* or SDI; iron deficiency and child growth failure, on the other hand, were higher in countries with low socioeconomic levels such as Malawi, India and Somalia. The factors were found to be substantially changing in countries undergoing rapid socioeconomic development, with the largest AAPC of suboptimal breastfeeding in Republic of Moldova [3.1% (2.9, 3.2)], child growth failure in Angola [−5.1% (−5.5, −4.6)], iron deficiency in Equatorial Guinea [−5.9% (−6.0, −5.7)], alcohol use in Vietnam [9.3% (8.4, 10.2)], and high BMI in Bosnia and Herzegovina [5.6% (5.3, 6.0)].

### Country-level associations of potential risk exposures in previous decades with incidence of EOCRC

**Figure 2:**
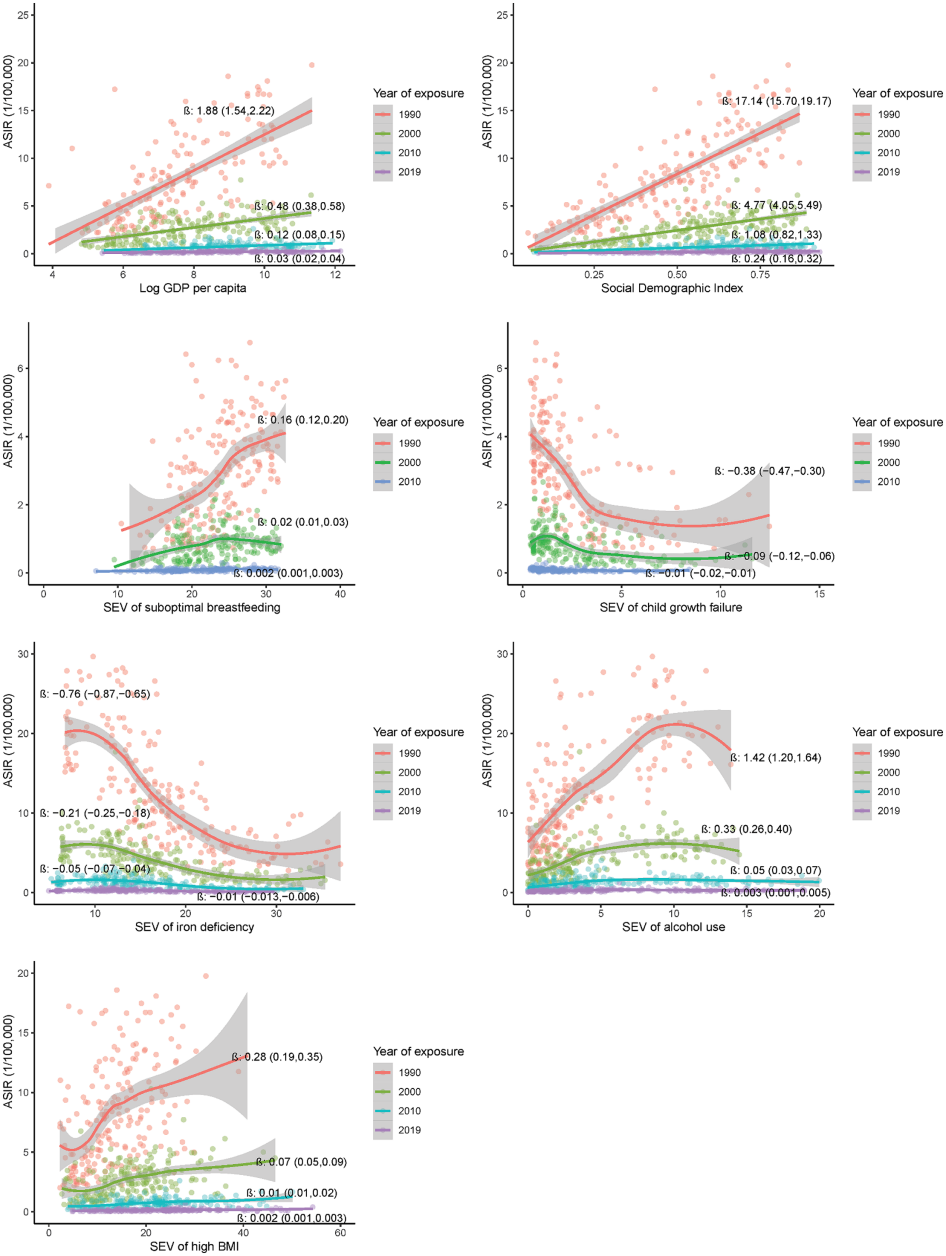
Country-level associations of selected risk exposures in 1990, 2000, 2010 and 2019 with the incidence of EOCRC in 2019.

As shown in Figure 2, a significant association was observed for GDP *per capita*, SDI, and SEVs of iron deficiency, alcohol use, high BMI, child growth failure and suboptimal breastfeeding in 1990, 2000, 2010 and 2019 with the incidence of EOCRC in 2019 at the country level [β (95%CI) ranging from −0.76 (−0.87, −0.65) to 17.14 (15.11, 19.18), all *p* values <0.001]. The significant associations were observed in both male and female populations (Supplementary Figure S7), and appeared more pronounced for the exposures in earlier calendar years than those in recent years. Similar association patterns were observed across the countries with high or low SDI levels (Supplementary Figure S8).

### Country-level associations of potential risk exposures at early age groups with incidence of EOCRC

**Figure 3:**
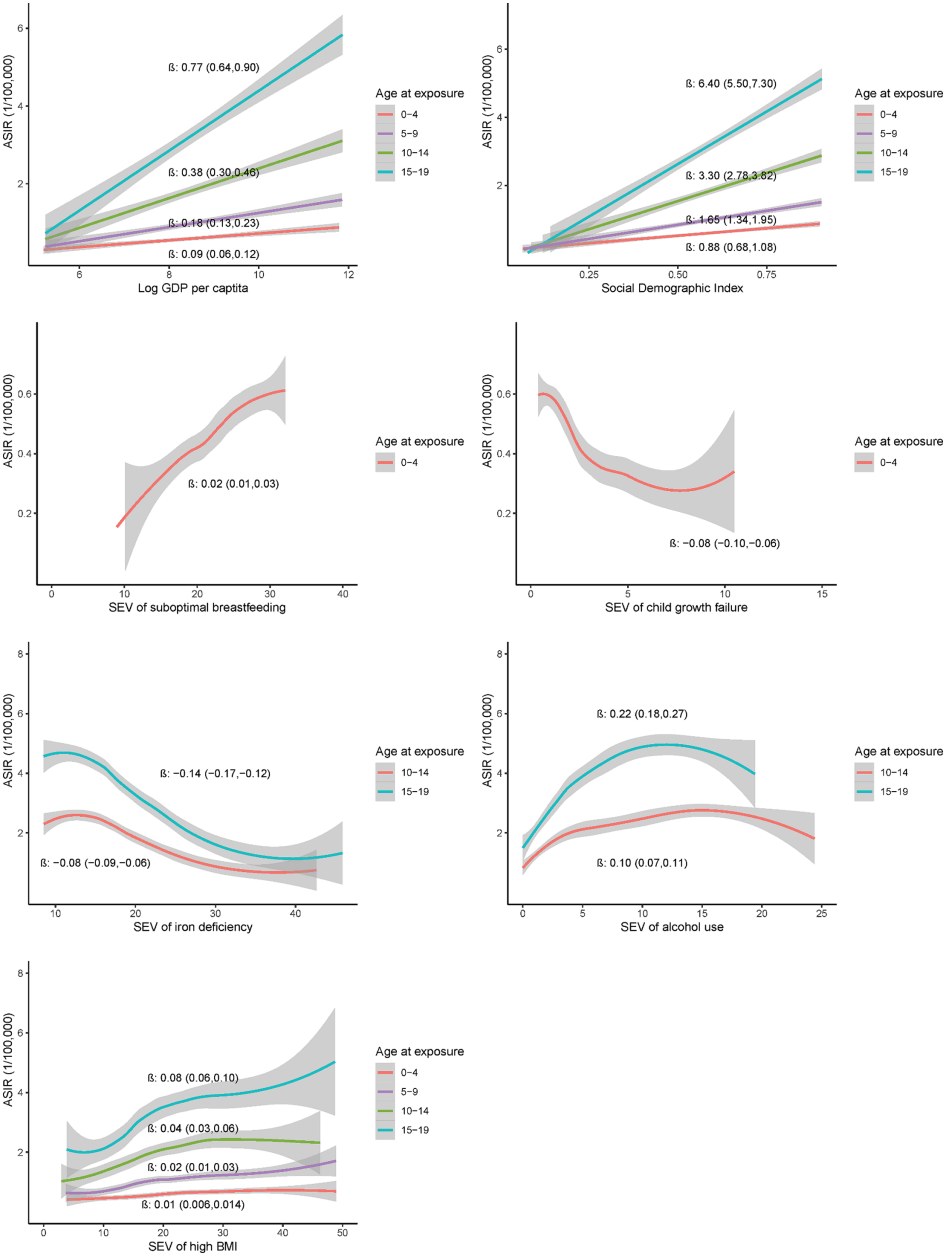
Country-level associations of selected risk exposures at 0–4, 5–9, 10–14 and 15–19 years with the incidence of EOCRC in 2019.

Further analysis on exposures at ages 0–4, 5–9, 10–14 and 15–19 years demonstrated significant associations of exposures at each age group with the incidence of EOCRC at the country level (Figure 3). The associations appeared more pronounced for the exposures at older age groups, with the largest β (95%CI) for GDP *per capita* [0.77 (0.64, 0.91)], SDI [6.40 (5.50, 7.30)], alcohol use [0.22 (0.18, 0.26)], high BMI [0.08 (0.06, 0.10)], and iron deficiency [−0.14 (−0.17, −0.12)] at 15–19 years (all *p* values less than 0.001). Similar association patterns were observed in the male and female populations (Supplementary Figure S9). The association patterns were also consistent across countries with high or low SDI (Supplementary Figure S10).

## Discussion

In this ecological analysis, we presented up-to-date results on the incidence of EOCRC and related exposures in early-life at national level. Consistent with previous studies (4, 31), we found a global increasing trend of EOCRC incidence over past three decades, particularly a higher incidence in countries or regions with a higher socioeconomic level and a substantially upward trend in countries or regions undergoing rapid socioeconomic development. Furthermore, we observed significant country-level associations of GDP *per capita*, SDI, and SEVs of iron deficiency, high BMI, suboptimal breastfeeding, child growth failure and alcohol use in early-life with the incidence of EOCRC. Our findings highlight the importance to prevent and control EOCRC in young populations, and indicate the potential contributions of the exposures in early-life to the large variations of EOCRC incidences across countries.

The higher incidence of EOCRC in the United States and European countries with a higher GDP *per capita* or SDI indicate higher risk exposures in the populations. The GDP *per capita* and the SDI represent the economic and social development level of a country or a region.

Generally, populations in highly developed countries or regions were more likely to have sedentary lifestyles, take more red meat and highly processed food, consume more cigarette and alcohol, and have higher prevalence of overweight or obesity, all of which have been recognized as risk factors of EOCRC (13, 32, 33). The drastically rising incidence in China and other countries experiencing more rapid socioeconomic development may also due to the increasing exposures to the risk factors due to epidemic of western lifestyle in these countries.

Since the GBD used the data from registry systems which have been better constructed in developed countries, the higher incidence of EOCRC in these countries may be attributed to a higher degree of completeness and accuracy of incidence reporting. Meanwhile, the fast-rising incidence in the countries experiencing a rapid development may be explained by the improving quality of cancer registries over the period. However, lower incidences were observed for stomach cancer and esophageal cancer in high-SDI regions than those with middle-SDI (34), and decreasing trends were found for stomach and liver cancers in developing countries, similar with the results based on the GLOBOCAN (35), both of which indicate the limited influence of the quality of registry data as well as modelling (36, 37).

Moreover, while the CRC screening is usually targeting the populations aged 50–74 years, the guidelines in the United States and many European countries recommended to lower the starting age from 50 to 45 years (30, 38), which may have improved the detection of colorectal lesions at the stage of polyps or adenoma and the early-stage of cancers in the young sub-populations. In the sensitivity analysis using the data of the US by including or excluding the population at age of 45–49 years, we did not observe substantially changed incidence and temporal pattern of EOCRC, probably due to the small coverage of screening and the double impacts of CRC screening on cancer incidence. Nevertheless, the unchanged incidence and its secular trend greatly mitigated our concern on the impact of CRC screening.

To explore the factors related with EOCRC, we defined the risk exposures in early life as those 10, 20 and 30 years ago or at ages 0–4, 5–9, 10–14 and 15–19 years. We found that suboptimal breastfeeding, child growth failure, iron deficiency, alcohol use and high BMI in previous decades or at the age groups were significantly associated with the incidences of EOCRC at the country level. The associations appeared more pronounced for the exposures in earlier calendar years and at ages 15–19 years, indicating the possibility that the risk exposures in early life, particularly during adolescence, may contribute to the development of EOCRC. Our results were consistent with previous studies based on the Nurses’ Health Study, in which exposure to risk factors like sugar-sweetened beverage and high BMI during adolescence were associated with a higher risk of EOCRC (20, 39).

The country-level associations of early-life exposure with EOCRC could be alternatively explained by the increased population coverage of the registry systems and improved quality of registry data over time. This may involve the lower-quality exposure data collected earlier and the higher quality incidence data collected later. If it was true, the associations of early-life exposures with subsequent EOCRC may have biased towards or away from null, which depended on whether the data were underestimated or overestimated. Furthermore, since SDI of each country may represent the quality of registration data and the degree of improvement in quality over time (24), the stratified analysis by SDI did not demonstrate different association patterns. Evidently, our results could not be fully explained by improved quality of data.

Of the five significant risk exposures, child growth failure and iron deficiency were higher in Low SDI regions, while higher suboptimal breastfeeding, alcohol use and high BMI were higher in High SDI regions, in line with the correlations of the factors with EOCRC incidences. Supporting evidence or potential mechanisms were also available for the factors with EOCRC. Suboptimal breastfeeding is an index for unbalanced nutrition in early life (under- nutrition or over-nutrition) due to replaced artificial feeding. The higher incidence of EOCRC in countries with a higher suboptimal breastfeeding was in line with the well-established protective effect of optimal breastfeeding practice. Breast milk has been suggested to reduce gastrointestinal inflammation and thus protect against development of ulcerative colitis (40). Children artificially fed could not benefit from the protections. Alcohol use is a widely-recognized risk factor for EOCRC and other diseases (41). It is of note that alcohol use varied greatly in young populations across countries, and demonstrated a reverse U-shaped association with incidence of EOCRC. Further analysis identified several East European countries as exceptional cases, which had the highest SEV of alcohol use but lower incidence of EOCRC, possibly due to the lower level of exposure to other risk factors (42). Heme iron involves in colorectal carcinogenesis through catalysing ROS production and changing intestinal microflora (43, 44). Since the iron deficiency was less prevalent in men (45), the higher incidence of EOCRC in men than in women was consistent with the negative ecological correlation of iron deficiency with EOCRC. Growth failure indicates insufficient nutrition in early life, which has been associated with lower risks of metabolic diseases and cancers in animals (46, 47). The country-level negative association of child growth failure with EOCRC in this study was consistent with a Dutch cohort study, in which growth failure was correlated with a lower risk of CRC at individual level (48). Contrary to growth failure, high BMI in early-life indicates excessive energy intake and represents abnormal metabolic status. High BMI or obesity in early-life has been associated with a higher risk of EOCRC in a case–control study (18) and a cohort study (20). In this study, a high BMI in early life, either defined as exposure in previous years or at ages 0 to 19 years, was consistently associated with a higher incidence of EOCRC at the country level. Our results indicate the possible contributions of nutrition and growth during childhood in subsequent EOCRC, which need to be confirmed in epidemiological studies at the individual level.

To the best of our knowledge, this analysis based on the *GBD 2019* was the first attempt to generate hypothesis that the risk exposures in early-life may involve in the development of EOCRC. The *GBD 2019* provided comprehensive and long-term data for estimates of global disease burden and related risk factors, based on which we could define the risk exposures in early life and ensure the evaluation of ecological correlations of the risk exposure with subsequent incidence of EOCRC in a same population at the country level.

However, there are several limitations in this study. First, as an ecological study, the associations at the country level could not support causal inference due to the possible ecological fallacy and potential confounding factors. For example, the close correlation of heme iron with red meat intake (41), the well-known risk factor for CRC (42), indicate the potential confounding effect of red meat intake. Therefore, the related exposures identified in this study just guide the generation of hypothesis on the potential risk factors for EOCRC. Second, to define the exposures in early life, we supposed that people in 1990 aged 0–19 years was exactly the same individuals aged 30–40 years in 2019, which might be assumptive in countries with low GDP *per capita* or huge movement of people. This may lead to misclassification bias. Nevertheless, the huge populations at region and country level may have minimized the potential bias. Finally, due to the inherent limitations of the *GBD 2019* data, we just focused on ten risk exposures in early-life, and were unable to evaluate other factors such as smoking, physical inactivity, intakes of red meat, processed meat, and whole grains with EOCRC.

## Conclusion

In summary, in this ecological analysis based on the *GBD 2019*, we observed a global fast-rising incidence of EOCRC and identified several exposures in early life associated with the incidences at the country level. Our results highlight the importance to prevent and control CRC in young populations, and help to generate hypothesis that the risk exposures during adolescence may contribute to the development of EOCRC.

## Data availability statement

Publicly available datasets were analyzed in this study. This data can be found at: https://vizhub.healthdata.org/gbd-results/.

## Author contributions

ZW: Formal analysis, Software, Writing – original draft. WY: Formal analysis, Writing – review & editing. WW: Formal analysis, Writing – review & editing. JH: Data curation, Writing – review & editing. YM: Supervision, Writing – review & editing. CY: Data curation, Writing – review & editing. JS: Supervision, Writing – review & editing. JF: Data curation, Writing – review & editing. YW: Data curation, Writing – review & editing. MW: Supervision, Writing – review & editing. WX: Conceptualization, Methodology, Supervision, Writing – review & editing.

## Footnotes

^1^https://data.worldbank.org/indicator/
