# Supplementary material for: Global incidence trends of early-onset colorectal cancer and related exposures in early-life: an ecological analysis based on the GBD 2019
Source: Front Public Health. 2024 Jun 20;12:1367818. doi: 10.3389/fpubh.2024.1367818 (PMC11222603; doi:10.3389/fpubh.2024.1367818)
Supplement: Supplementary file 1 [file Data_Sheet_1.PDF]

Supplementary content 1

Generally, SEV was calculated based on the following formula:

$$SEV = \frac{\int_{x=l}^{u} RR(x)\, P(x)\, d(x) - 1}{RRmax - 1}$$

Where RR(x) is a risk ratio at level x of exposure, RR_{max} is the highest risk ratio where more than 1% of population are exposed, P(x) is the density of exposure, and *l* and *u* are the lowest and the highest levels of exposure, respectively

Table S1: Adjusted β (95% CI) for selected risk exposures in early life with the incidence of EoCRC in 2019 at country level

SEV of risk factor	Mutually adjusted β (95% CI)	***P***-value	VIF
**By calendar year**			
In 1990			
Suboptimal breast feeding*	0.072 (0.021, 0.124)	<0.001	1.55
Child growth failure*	-0.296 (-0.399, -0.193)	<0.001	1.55
Alcohol use**	0.788 (0.527, 1.049)	<0.001	1.77
Iron deficiency**	-0.493 (-0.627, -0.360)	<0.001	1.77
In 2000			
Suboptimal breast feeding	-0.002 (-0.016, 0.012)	0.76	1.48
Child growth failure	-0.094 (-0.124, -0.063)	<0.001	1.48
Alcohol use	0.138 (0.056, 0.220)	<0.001	1.71
Iron deficiency	-0.163 (-0.208, -0.117)	<0.001	1.71
In 2010			
Suboptimal breast feeding	0.002 (-0.001, 0.004)	0.21	1.50
Child growth failure	-0.009 (-0.017, -0.001)	0.03	1.50
Alcohol use	0.003 (-0.017, 0.023)	0.76	1.63
Iron deficiency	-0.054 (-0.069, -0.040)	<0.001	1.63
In 2019			
Alcohol use	-0.002 (-0.007, 0.003)	0.358	1.60
Iron deficiency	-0.011 (-0.015, -0.007)	<0.001	1.60
**By age window**			
0-4 years			
Suboptimal breast feeding	0.001 (-0.010, 0.009)	0.81	1.59
Child growth failure	-0.064 (-0.095, -0.044)	<0.001	1.92
High BMI	0.004(-0.002, 0.008)	0.15	1.39
5-9 years			
High BMI	0.02 (0.01,0.03)	0.16	-
10-14 years			
Alcohol use	0.046 (0.023, 0.068)	<0.001	2.01
Iron deficiency	-0.045 (-0.066, -0.025)	<0.001	2.57
High BMI	0.014 (0.001, 0.277)	0.03	1.42
15-19 years			
Alcohol use	0.114 (0.066, 0.161)	<0.001	1.69
Iron deficiency	-0.090 (-0.121, -0.594)	<0.001	2.15
High BMI	0.023 (0.000, 0.457)	0.05	1.35

CI: Confidence Interval; VIF: Variance inflation factor

*Both suboptimal breastfeeding and child growth failure exposed at 0-4 years only, both iron deficiency and alcohol use exposed at 10-19 only

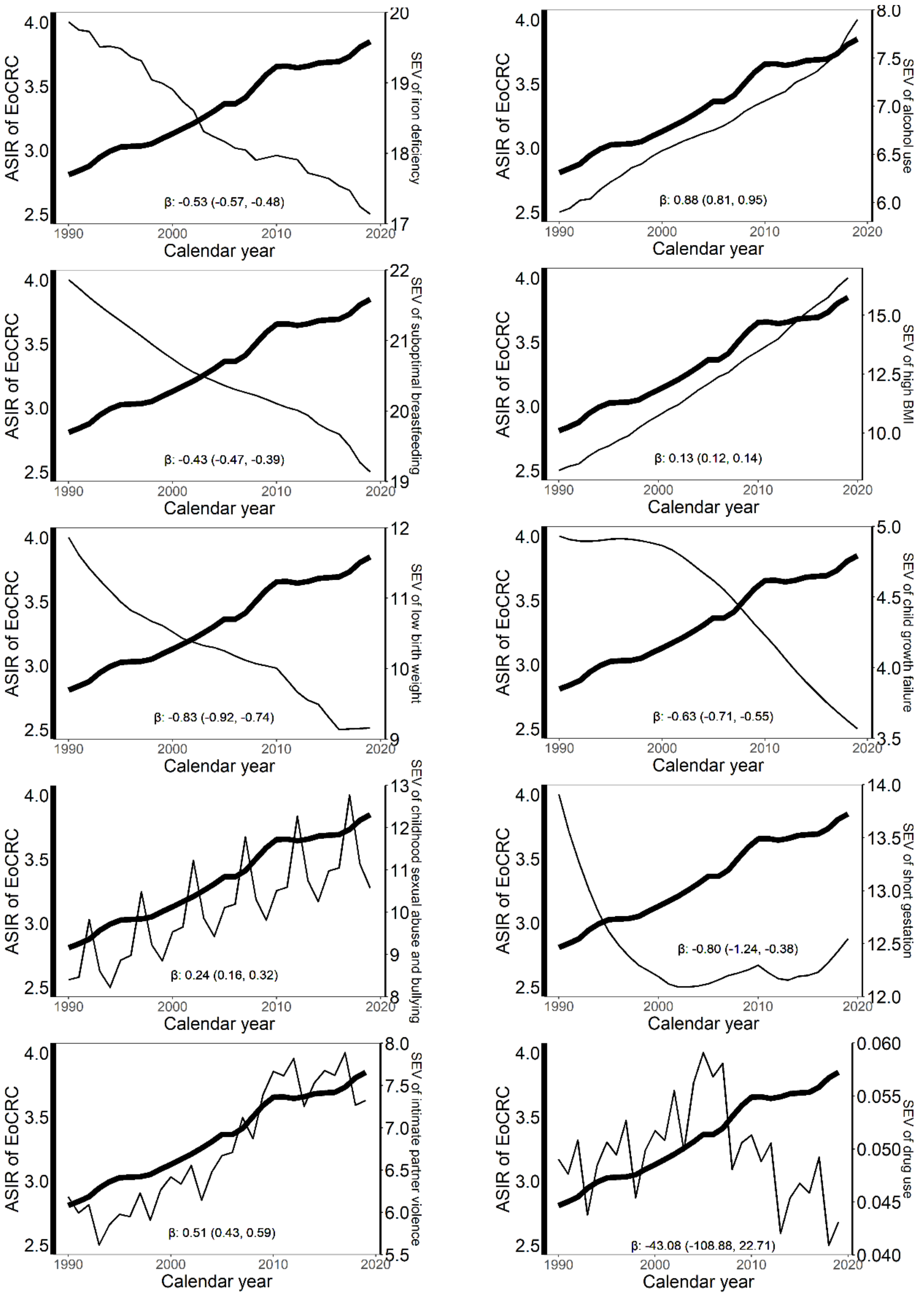

Figure S1: Global associations of risk factors in early life with the incidence of EoCRC over the period of 1990 and 2019

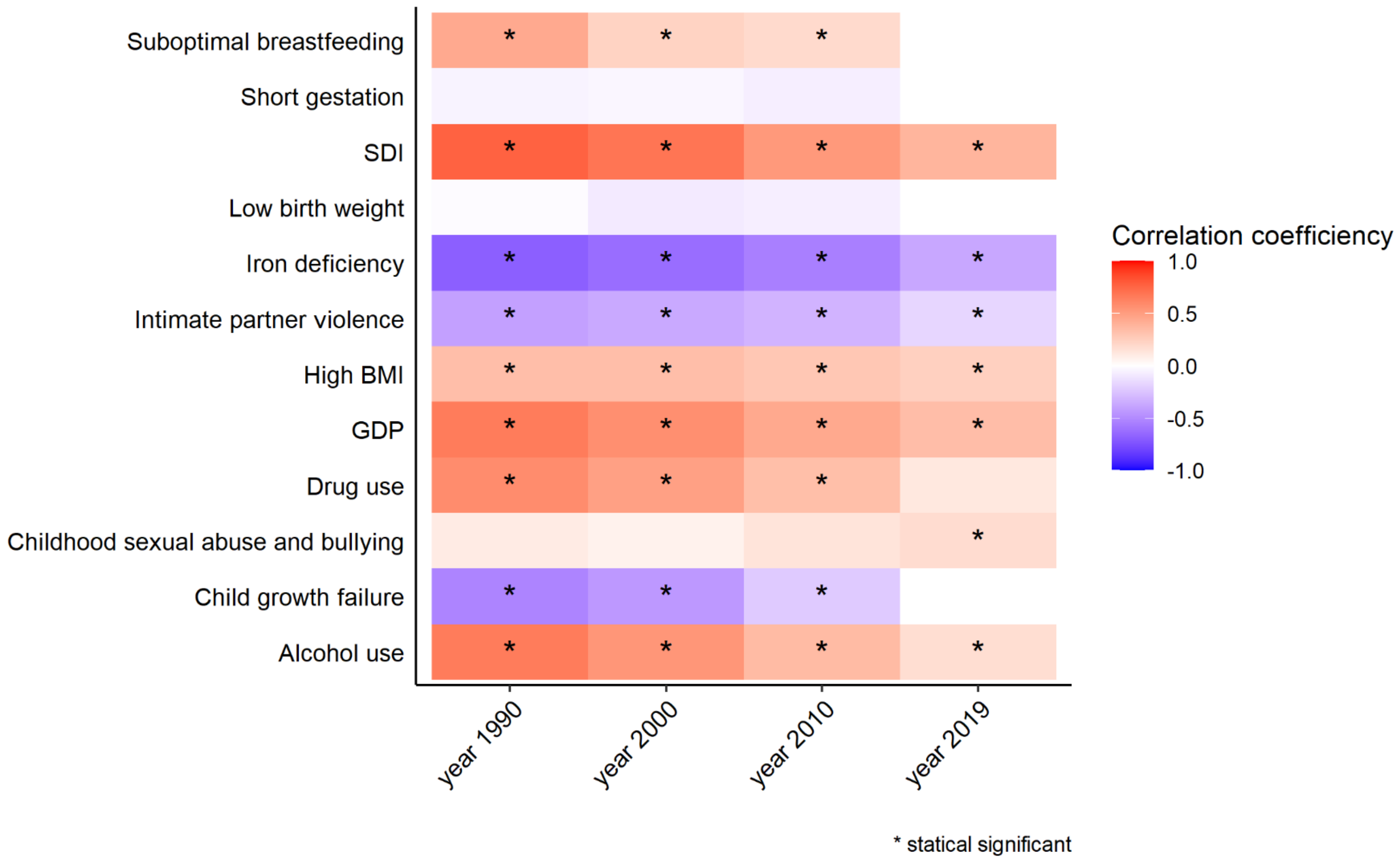

Figure S2: Correlation matrix of risk factors in early life with the incidence of EoCRC in 1990, 2000, 2010 and 2019 at country level

* $p < 0.05$.

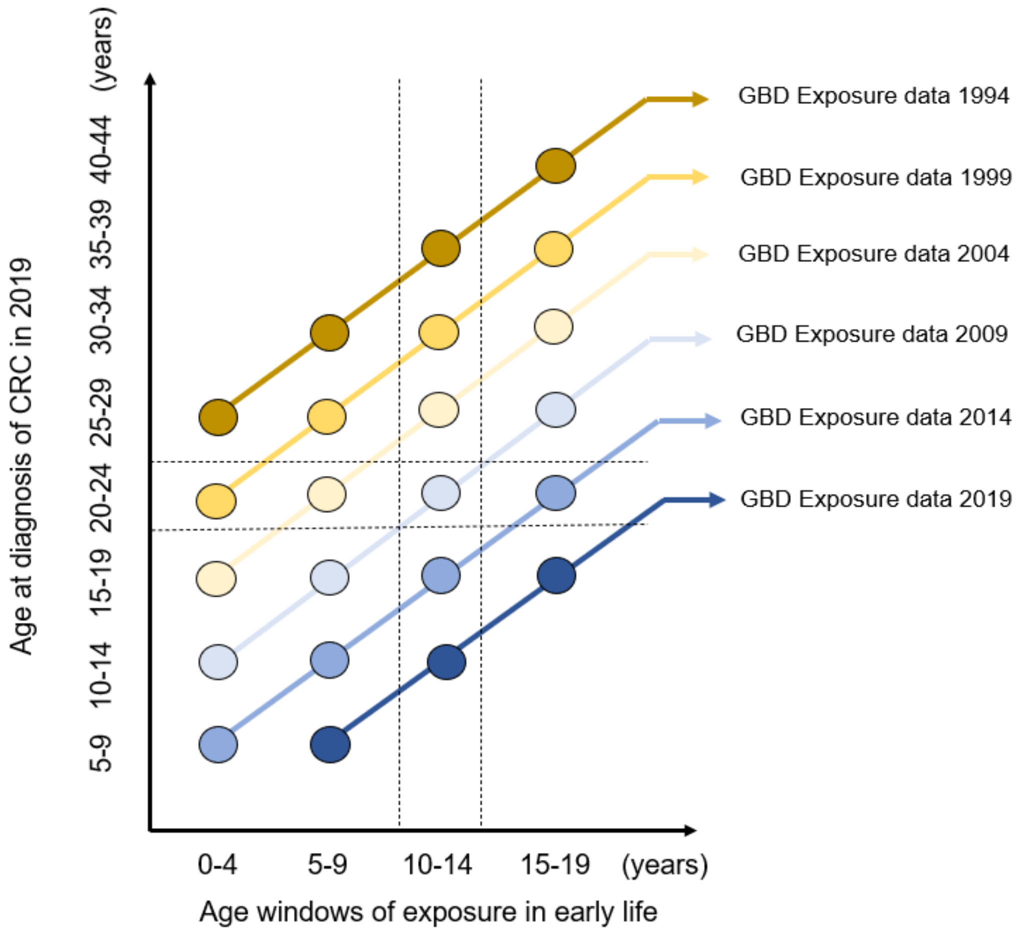

Figure S3: Schematic diagram for data extraction strategy on risk exposures at four age windows in early life

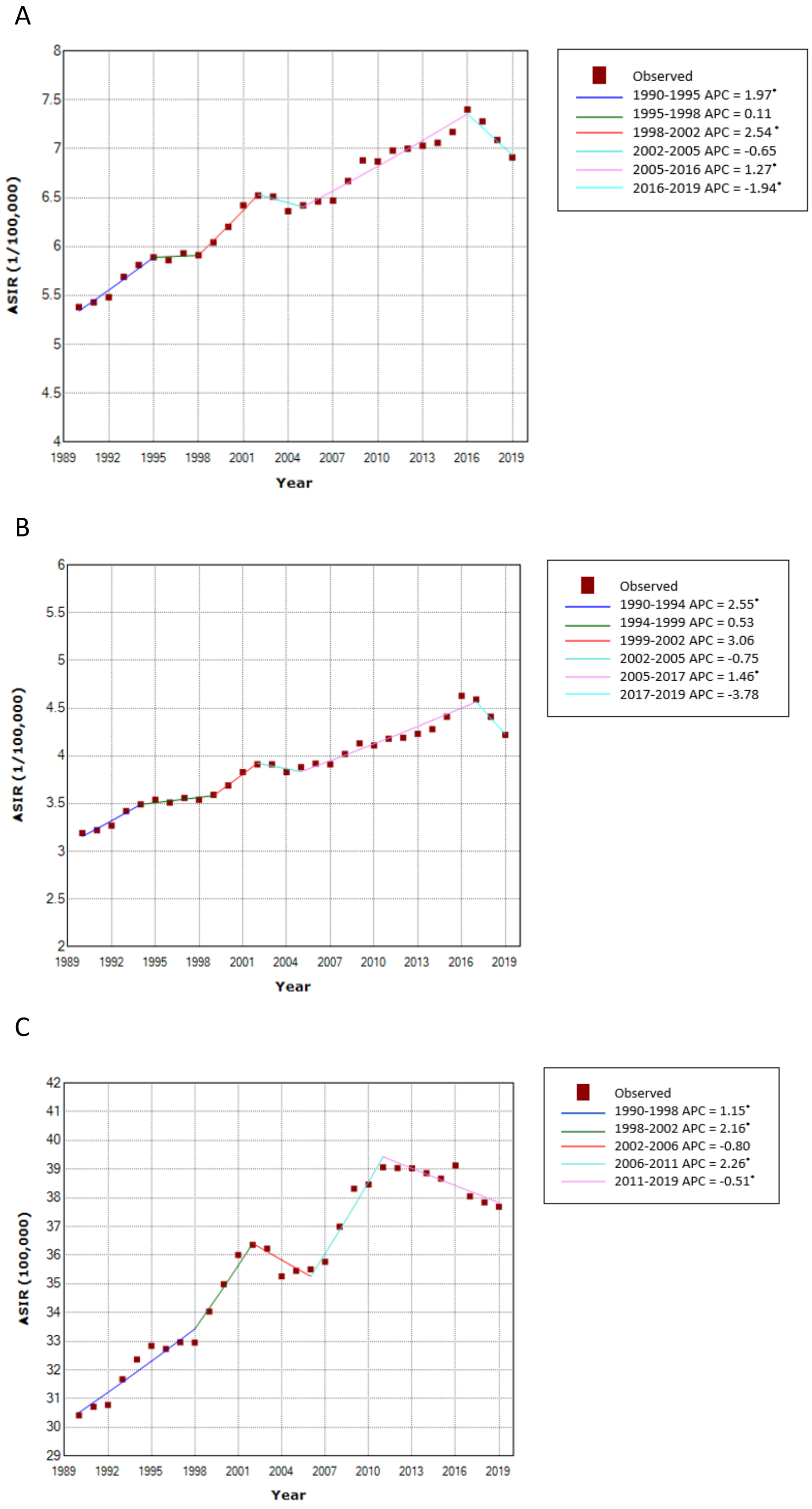

Figure S4: Sensitivity analysis of temporal patterns for incidence of EoCRC in the US. Joinpoint regression of ASR in population less than 50 years (A); in population less than 45 years (B); and in population at age of 45-49 years (C).

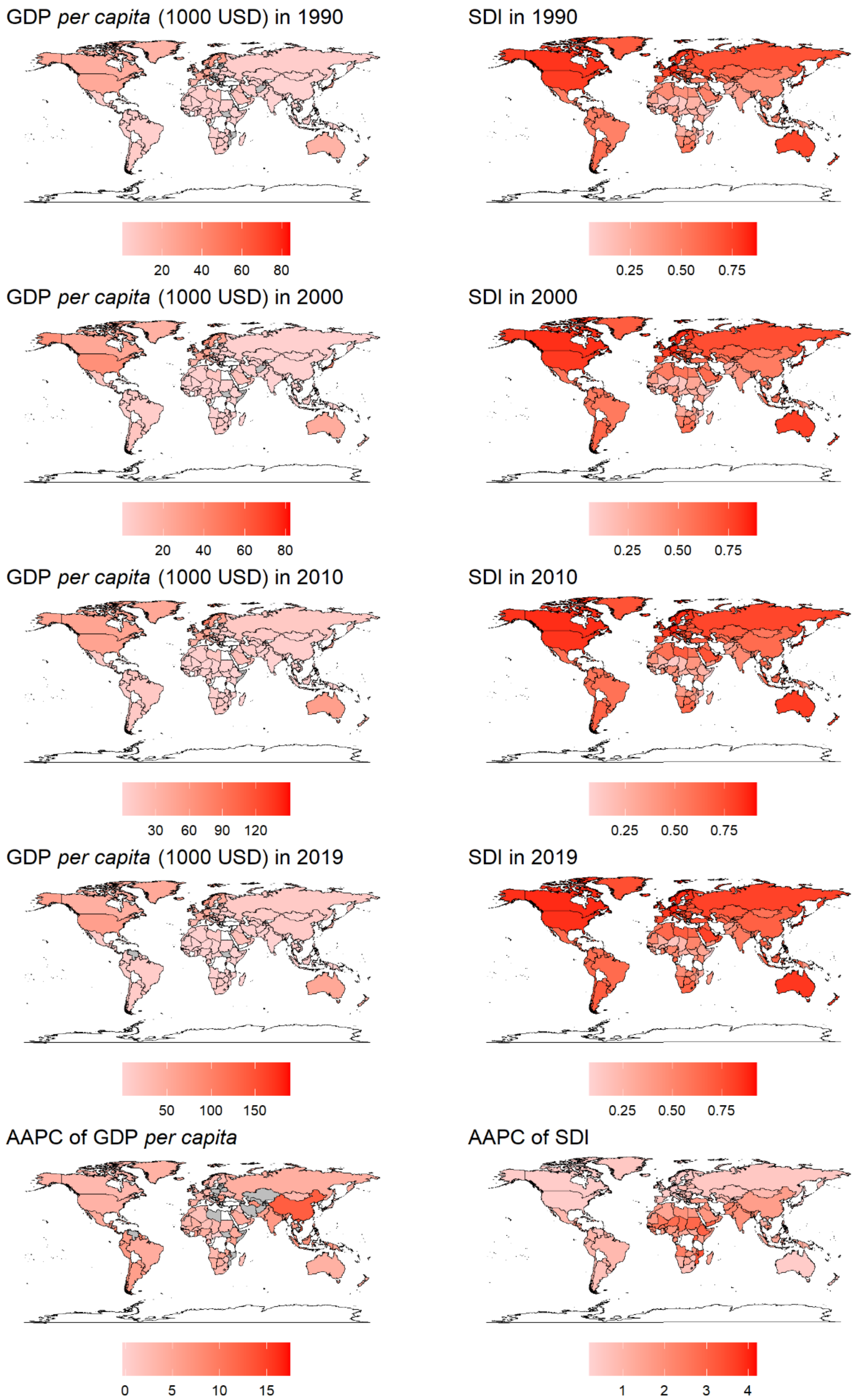

Figure S5. Country-level GDP *per capita* and SDI in 1990, 2000, 2010 and 2019 and AAPC from 1990 to 2019

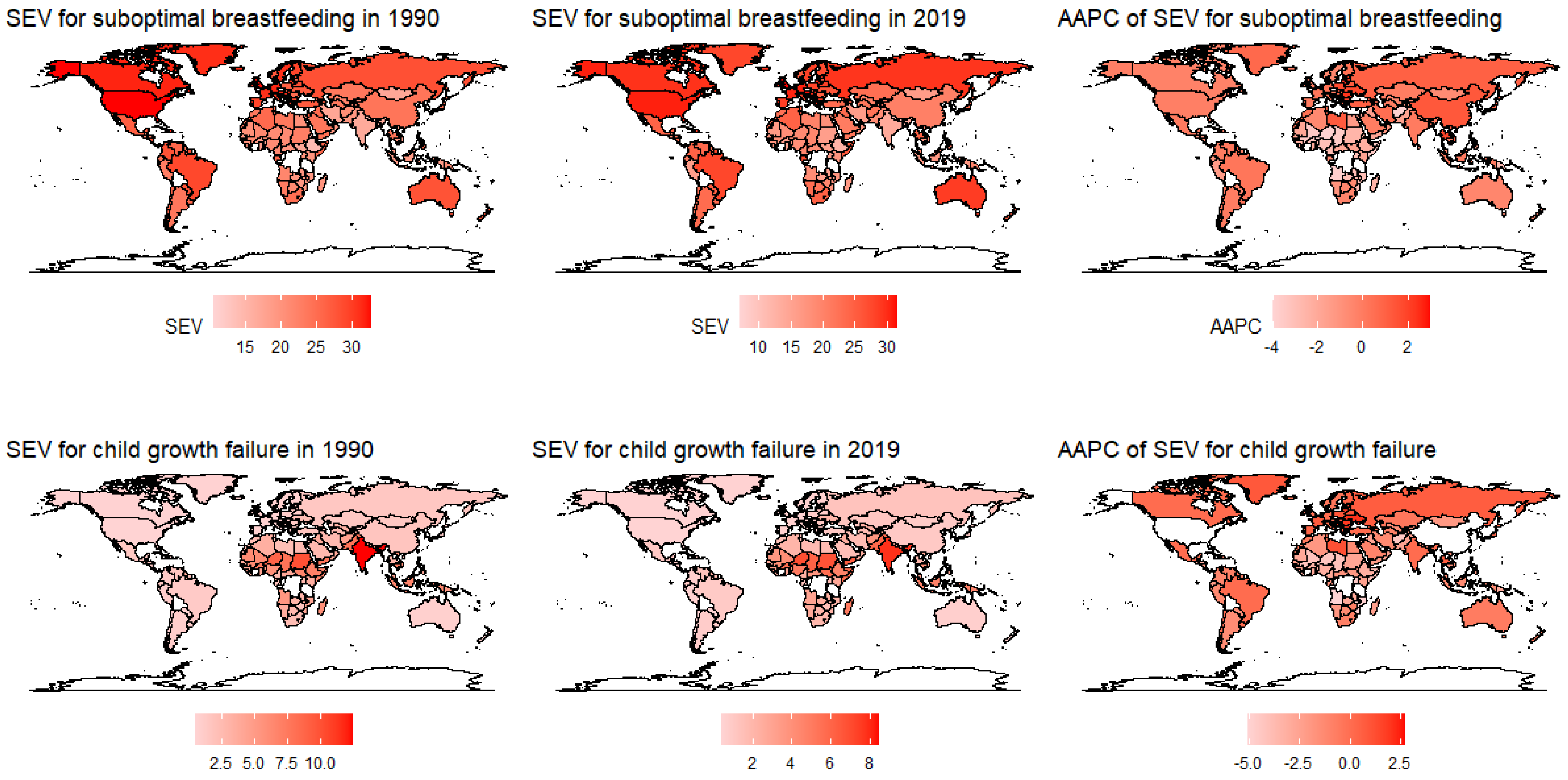
SEV for suboptimal breastfeeding in 1990
SEV
15 20 25 30
SEV for suboptimal breastfeeding in 2019
SEV
10 15 20 25 30
AAPC of SEV for suboptimal breastfeeding
AAPC
-4 -2 0 2
SEV for child growth failure in 1990
2.5 5.0 7.5 10.0
SEV for child growth failure in 2019
2 4 6 8
AAPC of SEV for child growth failure
-5.0 -2.5 0.0 2.5

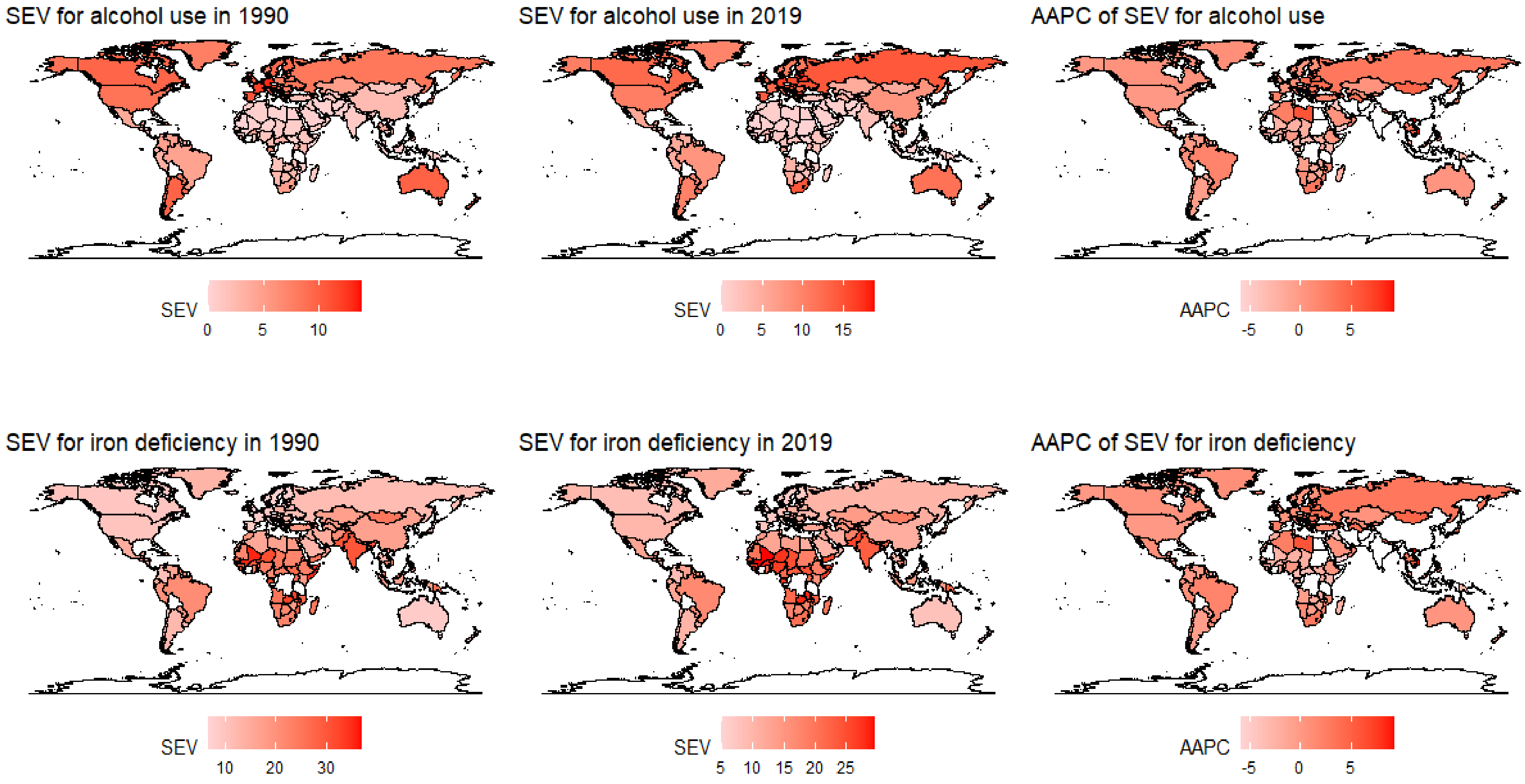
SEV for alcohol use in 1990
SEV
0 5 10
SEV for alcohol use in 2019
SEV
0 5 10 15
AAPC of SEV for alcohol use
AAPC
-5 0 5
SEV for iron deficiency in 1990
SEV
10 20 30
SEV for iron deficiency in 2019
SEV
5 10 15 20 25
AAPC of SEV for iron deficiency
AAPC
-5 0 5

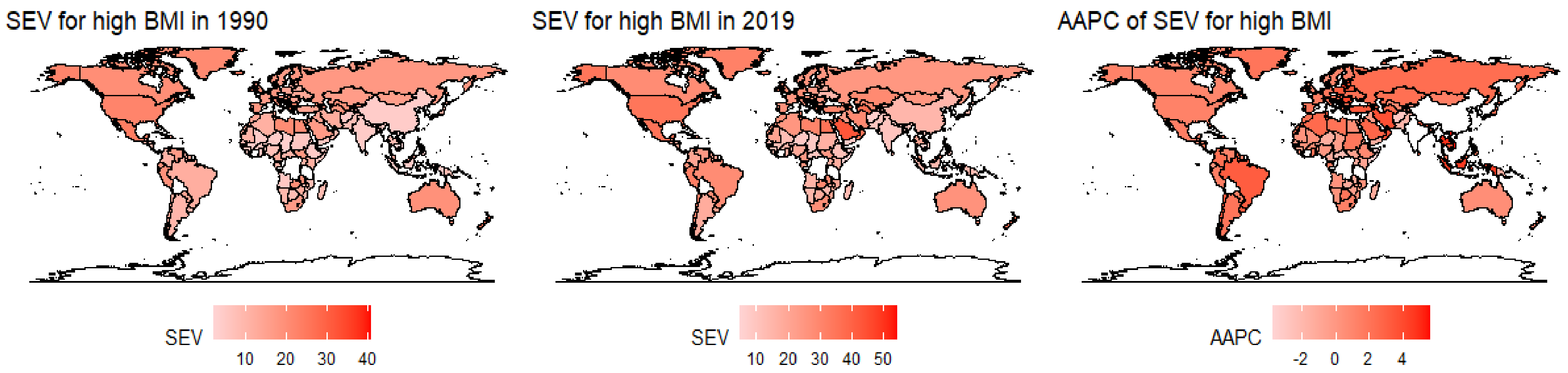

Figure S6: Summary exposure value for suboptimal breastfeeding (A), child growth failure (B), alcohol use (C), iron deficiency (D) and high body-mass index (E) in 1990, 2019 and AAPC at national level

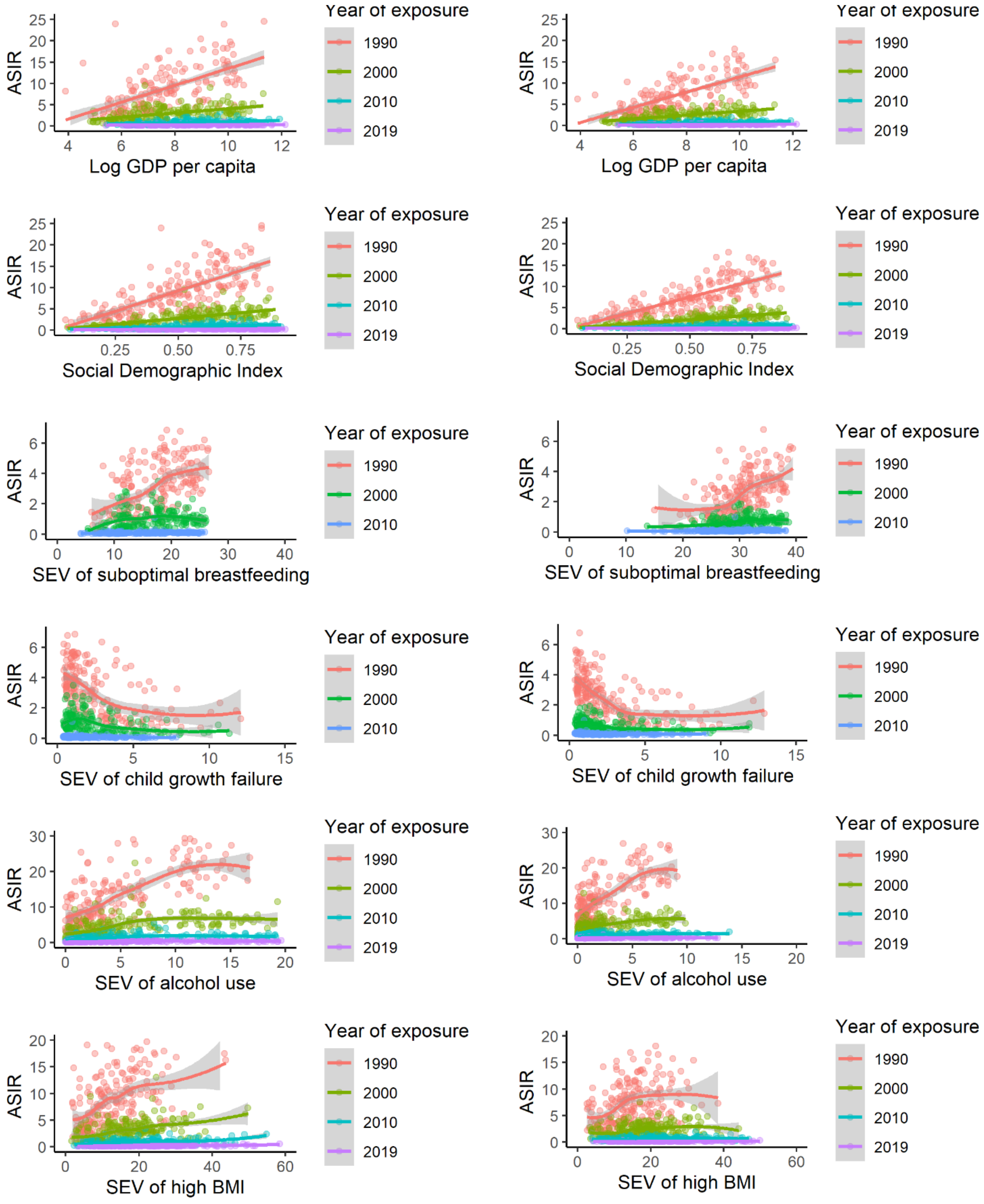

Figure S7: Associations of selected risk exposures in 1990, 2000, 2010 and 2019 with the incidence of EoCRC (1/100,000) in 2019 at national level in men (left) and women (right)

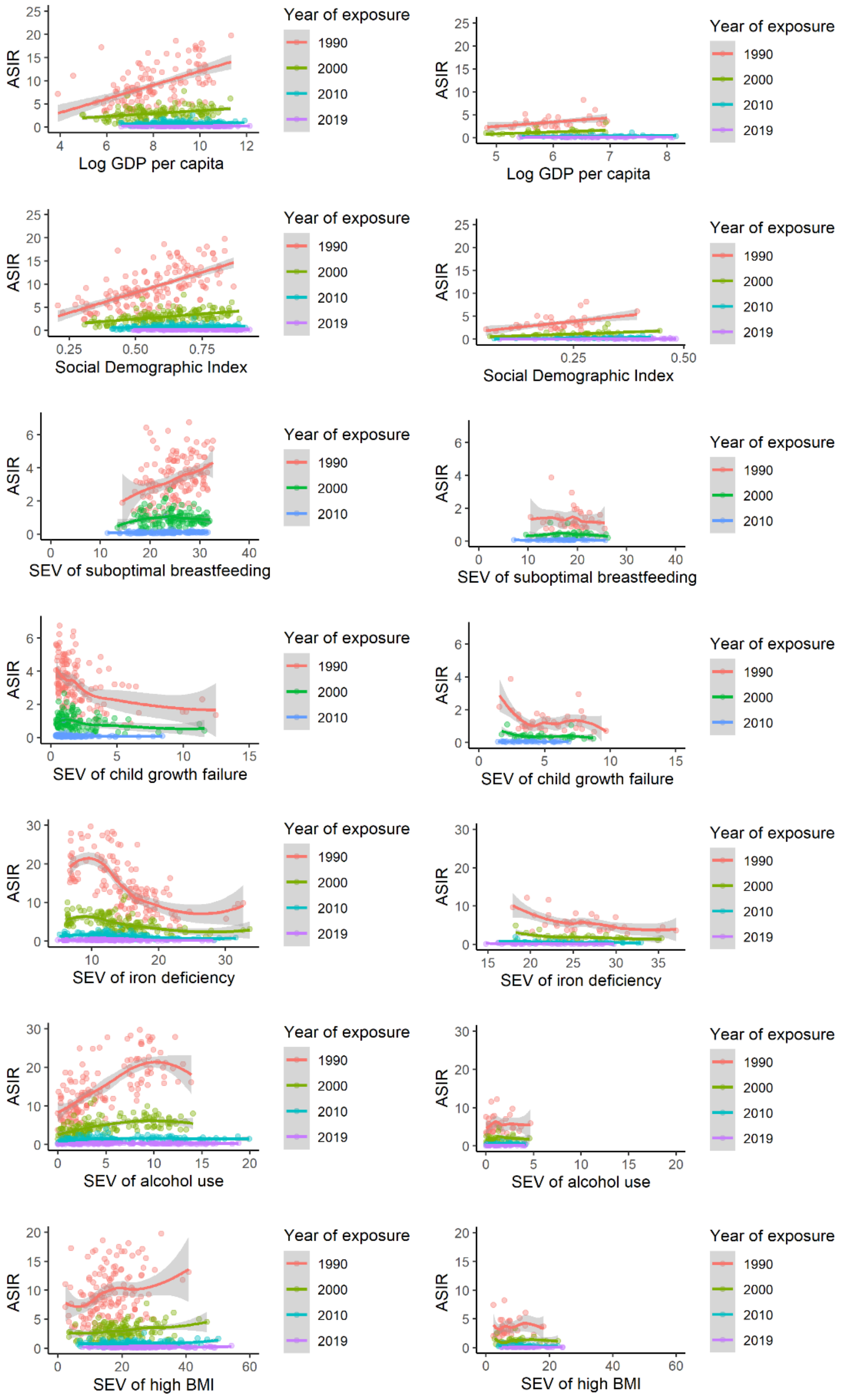

Figure S8: Associations of selected risk exposures in 1990, 2000, 2010 and 2019 with the incidence of EoCRC (1/100,000) in 2019 at national level across countries with high (left) or low SDI (right)

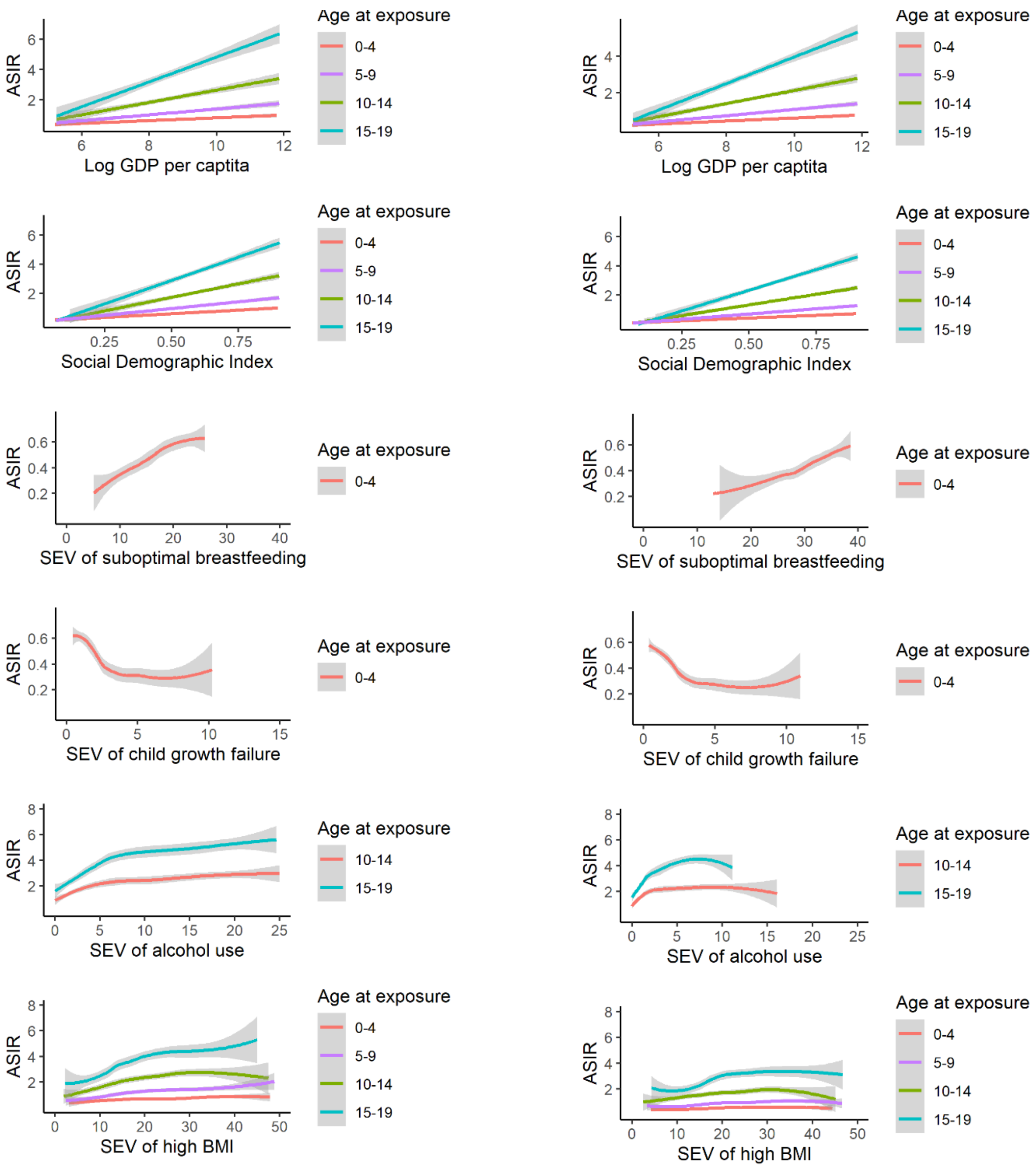

Figure S9: Associations of selected risk exposures at ages 0-4, 5-9, 10-14 and 15-19 years with the incidence of EoCRC (1/100,000) in 2019 at national level in male (left) and female populations (right)

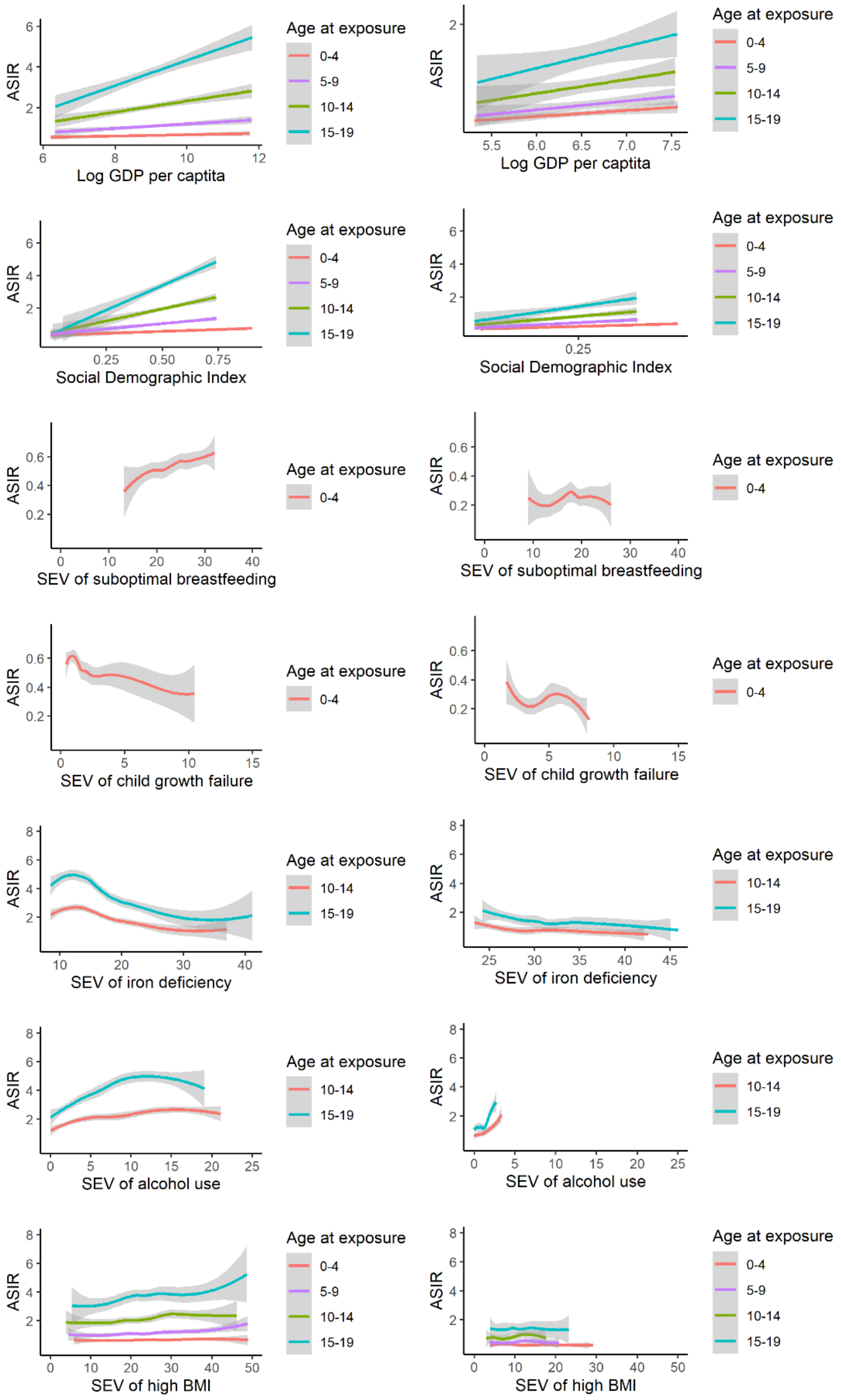

Figure S10: Associations of selected risk exposures at ages 0-4, 5-9, 10-14 and 15-19 years with the incidence of EoCRC (1/100,000) in 2019 at national level across countries with high (left) or low SDI (right)
